# Primary headaches, attention deficit disorder and learning disabilities in children and adolescents

**DOI:** 10.1186/1129-2377-14-54

**Published:** 2013-06-27

**Authors:** Jacob Genizi, Shiri Gordon, Nogah C Kerem, Isaac Srugo, Eli Shahar, Sarit Ravid

**Affiliations:** 1 Pediatric Department, Bnai Zion Medical Center, Haifa, Israel; 2 Pediatric Neurology Unit, Bnai Zion Medical Center, Haifa, Israel; 3 Adolescent Medicine Unit, Bnai Zion Medical Center, Haifa, Israel; 4 Pediatric Neurology Unit, Meyer Children’s Hospital, Rambam Medical Center, Haifa, Israel; 5 Bruce Rappaport Faculty of Medicine, Technion, Haifa, Israel

**Keywords:** Migraine, Tension type headache, Attention deficit disorder, Learning disabilities

## Abstract

**Background:**

Primary headaches and Learning difficulties are both common in the pediatric population. The goal of our study was to assess the prevalence of learning disabilities and attention deficit disorder in children and adolescents with migraine and tension type headaches.

**Methods:**

Retrospective review of medical records of children and adolescents who presented with headache to the outpatient pediatric neurology clinics of Bnai-Zion Medical Center and Meyer Children’s Hospital, Haifa, during the years 2009–2010. Demographics, Headache type, attention deficit disorder (ADHD), learning disabilities and academic achievements were assessed.

**Results:**

243 patients met the inclusion criteria and were assessed: 135 (55.6%) females and 108 (44.4%) males. 44% were diagnosed with migraine (35.8% of the males, 64.2% of the females, p = 0.04), 47.7% were diagnosed with tension type headache (50.4% of the males, 49.6% of the females). Among patients presenting with headache for the first time, 24% were formerly diagnosed with learning disabilities and 28% were diagnosed with attention deficit disorder (ADHD). ADHD was more prevalent among patients with tension type headache when compared with patients with migraine (36.5% vs. 19.8%, p = 0.006). Poor to average school academic performance was more prevalent among children with tension type headache, whereas good to excellent academic performance was more prevalent among those with migraine.

**Conclusions:**

Learning disabilities and ADHD are more common in children and adolescents who are referred for neurological assessment due to primary headaches than is described in the general pediatric population. There is an association between headache diagnosis and school achievements.

## Background

Primary headaches are common in the pediatric population (18.6–27.9%) [1,2], predominantly migraine and tension type headaches [3]. Since primary headaches may become disabling for children, several studies focused on the impact of headaches on school performance [4,5], however the impact was reported to be minimal for most children [6]. Attention deficit disorder (ADHD) and learning disabilities are also common among the pediatric population and are considered to be important factors leading to poor academic performance. The worldwide prevalence of ADHD is estimated to be 5.3%, [7] while in the United States the percentage is even higher in children aged 8 to 15 years-8% [8]. The prevalence of learning disabilities in the general population is estimated to be 2%–10%, depending on the type of evaluation and definition used. About 3–5% of students studying at U.S. public schools are considered to have learning disabilities [9].

Many studies in adults with migraine showed impairment in tasks such as psychomotor speed, attention, language [10,11] and executive functions [12], while others failed to confirm these findings [13]. In adults with tension type headache no cognitive dysfunction was identified [14]. The association between primary headaches and emotional and behavioral problems in children has been previously described [15,16], yet the prevalence of ADHD and learning disabilities among children with headaches as well as their association with headache duration and frequency is still contradictory. Concentration difficulties, emotional rigidity, deliberation and hyperactivity as well as stress among the family or at school are considered to be psychological predictors of headaches in the pediatric age group [17,18]. On the other hand frequent headaches may increase distractibility, particularly in children with primary short attention span and thus further insult learning.

The purpose of the present study was to assess the prevalence of learning disabilities and ADHD in children and adolescents with primary headaches, and to examine their relations to the type, the frequency and the duration of the headaches. We hypothesized that more frequent headaches would be associated with a high prevalence of learning disabilities, low academic achievements, and ADHD in children.

## Methods

### Patients

Children and adolescents aged 6–18 years, who were referred by their primary physician for neurological assessment due to headaches to the outpatient pediatric neurology clinics at Bnai-Zion Medical Center and at Meyer Children’s Hospital, Haifa, during the years 2009–2010.

### Inclusion criteria

Children and adolescents aged 6–18 years, that met the diagnostic criteria for either migraine or tension type headache (TTH) according to the International Headache Society [19], whose parents signed an informed consent form.

### Exclusion criteria

Children younger than 6 years, children with developmental delay, mental retardation, seizure disorder, major psychiatric disorders, hydrocephalus or any other systemic condition that may have an impact on headaches and brain function.

### Study procedure

After the Rambam Medical Center IRB's approval (#0115-11) was obtained, a retrospective review of medical records was carried out. During their initial and follow up visits to the pediatric neurology clinic all patients and parents were interviewed using a semi-structured interview and were asked to answer a written questionnaire. Both the interview and the questionnaire included questions regarding demographics, patient’s and family medical history, headaches history (age at onset, location, quality, frequency, duration of episodes, aura, associating symptoms). Parents and patients have evaluated their school performance as poor (40–50/100), average (60–70/100), good (80–90/100) and excellent (90/100). Patients were subdivided into groups according to headache episodes’ duration and frequency. The division for duration was: A-less than 2 hours, B-2 to 4 hours, C-more than 4 hours, and for frequency: A-less than 4 episodes per month, B-five to ten episodes per month, C-more than 10 episodes per month.

Children were diagnosed as ADHD if they fulfilled the DSM-4 criteria [20] using the Conners’ Parents and Teacher Rating Scales-Revised [21]. This test is typically used to measure symptoms of ADHD (hyperactivity, inattention, and impulsivity). The diagnosis of learning disabilities was based on DSM-4 criteria [20] and formal psycho-educational assessment.

The association between headache duration and frequency, ADHD, learning disabilities and academic achievements was assessed.

### Statistical evaluation

Data was summarized as proportions or means and standard deviations. Data was analyzed using standard statistical methods. Bivariate analyses of the associations between headache diagnosis, frequency and duration of headache and background factors (gender, learning disabilities, ADHD and school functioning) were conducted with Mann–Whitney U tests. Multivariate analyses of the associations between headache diagnosis and learning disabilities, ADHD and school functioning, were conducted with nominal regressions. Multivariate analyses of the associations between frequency and duration of headache and learning disabilities, ADHD and school functioning, were conducted with logistic regressions. Analyses for the total sample, in both nominal and logistic regressions, were adjusted for age and gender. No adjustment for multiplicity was made to the alpha levels of the statistical tests.

## Results

During the study period, a total of 243 children aged 6–18 years (Mean 11.24, SD = 3.08) were diagnosed with primary headache, 44.4% were males (108) and 55.6% females (135). Headache diagnosis was made according to the 2004 ICHD-II criteria [6]: 44% (107) were diagnosed with migraine (35.8% of the boys, 64.2% of the girls, p = 0.04). 47.7% (116) were diagnosed with tension type headache (50.4% of the boys, 49.6% of the girls). In the remaining 20 patients headache features did not fit into the ICHD-II criteria. Approximately 27.5% (66) had less than four episodes per month, 45% (108) had between 4–10 episodes each month and 27.5% (66) of the patients experienced more than 10 episodes of headache each month. In 43.3% (104) of the children headache episodes were described as short, lasting less than 2 hours, and 22.9% (55) of the patients described episodes that lasted more than four hours (Table 1).

**Table 1:** Headache diagnosis, frequency and duration

		**Total**	**Boys**	**Girls**	**p**
		**N (%)**	**N (%)**	**N (%)**	
Headache frequency	< 4 per month	66 (27.5%)	31 (47%)	35 (53%)	NS
	4–10 per month	108 (45%)	43 (39.8%)	65 (60.2%)	
	>10 per month	66 (27.5%)	33 (50%)	33 (50%)	
Headache duration	< 2 hours	104 (43.3%)	44 (42.3%)	60 (57.7%)	NS
	2–4 hours	81 (33.8%)	39 (48.1%)	42 (51.9%)	
	>4 hours	55 (22.9%)	24 (43.6%)	31 (56.4%)	

Overall 40.7% of children had either learning disabilities, or ADHD, or both. High rates of learning disabilities (24.7%), and ADHD (28%) were found in our study group. These rates are much higher than what is reported for the general population of Israeli children–10% for learning disabilities and 5–10% for ADHD (p < 0.05) [22]. ADHD was more prevalent among children with tension type headache compared to migraine (36.5% vs. 19.8%, p = 0.006). Poor and average school performance were more prevalent among children with tension type headache when compared to children with migraine (45% Vs. 26%, p < 0.005), whereas good to excellent school performance was more prevalent among those with migraine when compared to patients with tension type headache (74% Vs. 55%, p < 0.005), (Table 2).

**Table 2:** The association between: headache type, learning disabilities, ADHD and school performance

	**Learning disabilities**	ADHD**	**School performance**	
			**Good/Excellent**	**Poor/Average**
Migraine	28 (26.4%)	21 (19.8%)	27 (26.2%)	76 (73.8%)
TTH*	25 (21.7%)	42 (36.5%)	51 (44.7%)	63 (55.3%)
P	0.417	0.006	0.005	

*TTH Tension Type Headache.

**ADHD Attention deficit disorder.

### Associations between headache frequency, duration and learning disabilities and school performance

Frequency of headache episodes was found to be associated with lower academic achievements. Poor to average school performance was more prevalent among children with more than 10 episodes of headache per month compared to children with fewer headache episodes (48.5% vs. 33.7%, p = 0.032). The same was found for low academic achievements, they were more prevalent among those with more than 10 headache attacks per month when compared to patients with less frequent episodes (OR 2.00, CI-1.01–3.97, p = 0.047).

Duration of headache episodes was not found to be related to gender, ADHD or academic performance. However, children with headache episodes that lasted over 4 hours had more learning disabilities and lower grades at school than did children with shorter duration of headaches (p = 0.027, and p = 0.042, respectively).

## Discussion

The main findings in our study were the high rates of learning disabilities in children referred to our pediatric neurology clinics due to recurrent episodes of headaches: 24.7% had learning disabilities, 28% had ADHD, and 12% had both.

### Attention deficit disorder and headache

Leviton [23] was one of the first to report that out of 150 elementary-school children who were referred to his clinic due to recurrent headaches, approximately 40% had academic difficulties. These results are also in accordance with other recent studies that found a significant higher incidence of hyperactivity and impulsivity symptoms in children with headache, compared with healthy peers [11,24]. In a population-based study on healthy 4–17-years old children that was conducted by Strine et al. [10], children with frequent headaches were 2.6 times more likely to have inattention and hyperactivity. Yet, the association between ADHD and headache type remains controversial. Villa et. al. [25] described impaired visual attention in children with migraine, and suggested that it depends on neurotransmitters such as dopamine and noradrenalin. These same neurotransmitters are involved in the pathophysiology of migraine; therefore they suggested it may dispose those children to attention deficit. In recent studies by Riva [26,27] a significant association between attention related problems and headaches was demonstrated in both conditions-migraine and tension type headache. Some authors suggested that migraine and tension headache form a continuum that may share the same pathophysiological mechanisms and that the cerebral circuits subserving headache, personality profile, and attention-may overlap [28].

In accordance with other studies [16] we found a higher incidence of ADHD and lower school achievements in children with tension type headache in comparison with children with migraine. One possible explanation is that low school achievements and symptoms such as inattention, hyperactivity and impulsivity, which often accompany learning difficulties, may be associated with stress in the family, with peers, and in school, each stress may in turn contribute to symptoms of tension type headache.

A different hypothesis is that frequent headaches by themselves may increase distractibility and irritation especially in children with a primary short attention span, and thus impose a further insult on the challenge of learning. A third hypothesis is that a common disorder underlies both conditions: ADHD and headaches. The two latter theories are supported by a study that found correlation between neuropsychological defects and frequency of headache episodes [29]. In addition, children with frequent, severe headaches were more likely to exhibit high level of emotional, conduct, attention, language and peer problems, and were significantly more likely to have their disabilities interfere classroom learning and leisure activities [15]. However one should note, that since ADHD is more prevalent in boys than in girls, and since there were more boys in the group with tension type headaches-it is expected that ADHD would be more prevalent in the TTH group. Yet the gender difference was not enough to statistically explain the difference between the migraine and the TTH groups regarding ADHD prevalence and low school performance.

### Learning disorders and headache

Higher rates of learning disabilities (24.7%) were found in our study group compared with the reported rates in the general population of Israel [22]. Learning disabilities were more prevalent in children with migraine compared to children with tension type headache, in children with long duration of headache and among children with more than 10 episodes of headache per month. Only few studies addressed the relations between learning disabilities and headaches in children. D’Andrea and Waldie [30,31] both reported impairment in memory, visual-motor integration and poor verbal abilities, with normal performance in reading, arithmetic or motor and spatial tasks in children with migraine. Haverkamp compared the cognitive performance of children with migraine to their healthy siblings, and found no significant difference in sequential and simultaneous information processing [32]. A possible explanation to our findings is the theory of fear of failure that was found in children with chronic headaches, and as a result an over achievement approach to school work. Greater motivation to achieve has been reported in adolescents with headaches, with a positive interaction between desire for successes and achievements [33].

### Study limitation

Our study has a few limitations: It was conducted on a clinical sample of children with headaches, meaning children who were referred to a specialized neurology clinic, and not on a sample of healthy children (like a school based study) that would identify children with headaches and then look into different aspects of their learning. According to Berkson’s principal [34] people who seek medical care are more likely to have more than one medical problem, therefore the relationship between two diseases should not be studied in such a population. Thus we speculate that the rates of ADHD and learning disabilities that were found in our study may be applicable to those children with headaches who seek medical care, but may be an overestimation for children with headaches that are not treated in a specialized medical facility. This limitation should not impact the relations found between headache diagnosis, characteristics and learning challenges (ADHD and learning disabilities) that were indicated in our study. More limitations to our study are the lack of a formal psychiatric assessment (though children with known psychiatric disorders were excluded from the study), and the wide age span of our subjects.

## Conclusion

Our study demonstrates that there is an association between headaches, ADHD, learning disabilities, and school performance. Thus, taking a thorough history relating to all those parameters is essential when evaluating a child or an adolescent with primary headaches. Early diagnosis and treatment of ADHD and learning disabilities may improve school performance and thus the child’s well-being. Consequently there might be a positive effect on the reduction of headache episode. It is yet to be evaluated whether a better control of headaches will have a positive impact on school performance. In order to better understand the complicated relationship between headaches, ADHD, learning disabilities and school performance a large scale study should be conducted on a non-clinical population of children and adolescents, with a distinction between the two age groups.

## Competing interests

None of the authors has any conflict of interest to disclose.

## Authors’ contributions

JG SG & SR conceived the study, and participated in its design and coordination. JG wrote the first draft. NC ES participated in the design of the study and helped to draft the manuscript. All authors read and approved the final manuscript.
